# The applicability of quality improvement research for comparative effectiveness

**DOI:** 10.1186/1748-5908-8-S1-S6

**Published:** 2013-04-19

**Authors:** Erin E Redle, David Atkins

**Affiliations:** 1 Division of Speech Pathology, Cincinnati Children’s Hospital Medical Center, Cincinnati, Ohio, 45229, USA; 2 Department of Communication Sciences and Disorders, University of Cincinnati, Cincinnati, Ohio, 45229, USA; 3 Health Services Research and Development, Veterans Health Administration, Washington, District of Columbia, 20420, USA

## Presentation

Quality improvement research (QIR) and comparative effectiveness research (CER) share a common goal of achieving a higher-performing healthcare system. Both CER and QIR have grown considerably over the past decade, CER out of the need for more relevant information for clinical and policy decisions, QIR as a result of the increasing attention to the uneven quality of healthcare [1]. Although the two fields have often favored different research methods – CER often relies on direct, controlled experimental comparisons while QIR often favors single arm studies in a “real world” context, both of these methodologies are needed for improving patient care. Future studies that incorporate elements of both disciplines will provide a context for understanding the most effective and efficient methods for changing clinical practice and ultimately improving patient outcomes.

Better comparative methods for QIR would allow us to select the best quality improvement strategies for a given clinical setting. All QI interventions have costs (including opportunity costs) and none work in all settings and circumstances. As an example, quality improvement methods within the Veterans Administration (VA) have employed a variety of approaches which individually have evidence of effectiveness. These include:

• Provider education

• Patient education and support

• Electronic health records with clinical reminders or clinical decision support

• National formulary policies

• Performance measurement and reporting

• System re-engineering approaches and practice redesign

• Patient registries

• Change initiatives using collaboratives, champions, and toolkits

• Provider and management incentives

What we don’t know, however, is which interventions or bundles of interventions are most effective and efficient, with fewest harms or unintended effects, for specific quality improvement aims. Clinical reminders, for example, are easy to implement but their effectiveness varies and overusing reminders can lead to “reminder fatigue” and clinician resentment. In an era when front-line clinicians are feeling pressed by increasing responsibility and decreasing time, it is critical to match QI interventions to the specific needs [2].

Reliable comparisons of QI interventions will also require more complete descriptions of the context in which the improvement efforts are being undertaken. For example, although we know provider incentives can be effective tools for changing provider behavior, we don’t know the best ways to target them or how to set the right level of incentive. Recent work compared incentives aimed at physician groups vs. individuals and incentives targeting clinical teams vs. only physicians [3]. Understanding the context in which individuals are more or less responsive to incentives would inform future implementation efforts. QIR would also benefit from focusing attention on the marginal benefits of additional elements of QI interventions (for example, adding performance measurement to system re-engineering approaches), the unintended consequences of QI interventions, and the more complete assessment of the budget impact and business case for specific QI strategies.

## Commentary

At the same time, quality improvement research and implementation science are critical to ensuring that our current investment in CER will actually yield the intended gains in quality and value of health care. Simply disseminating CER findings is unlikely to change practice. Getting from high-quality clinical evidence to reliable and effective practice – what Dougherty has termed the T3 translation step – requires addressing the barriers and facilitators identified from implementation research [4]. These include patient and provider expectations and skills; financial and other incentives; leadership support and resources; availability of useful data; and role of opinion leaders or facilitators. To take one example, despite CER suggesting that conservative therapy without imaging is appropriate for most patients with acute low back pain [5], changing practice may require changing the financial incentives under which hospitals make more money performing diagnostic imaging and surgery than by providing physical therapy and follow-up.

A final lesson from QIR is that CER studies should be designed with implementation in mind. This may involve considering feasibility, potential for spread and the business case at the beginning when deciding what approaches are worth comparing. Incorporating qualitative methods into CER studies can yield important insights for learning how to spread and sustain whichever interventions prove to be effective. Hybrid studies are an important tool to build evidence of effectiveness for new interventions while also studying the implementation process [6].

## Recommendations

Both CER and QIR must overcome four challenges if they are to achieve the goal of contributing to reliable, high quality, high value healthcare. First, they must balance the desires for achieving greater relevance while maintaining adequate scientific rigor. Learning about what works in everyday practice and in typical patients will require us to move beyond those questions we can ask in prospective, experimental studies. We need to learn how to extract reliable information about both comparative effectiveness and quality improvement from the growing body of clinical data available from electronic health records; new methods such as natural language processing are expanding the range of clinical data that can be extracted from large data sets. Similarly, we need to improve our ability to draw valid inferences from the numerous “natural experiments” occurring in practice as health systems and individual practices adopt new approaches to problems of costs, quality, safety or patient satisfaction. This will require continuing to refine methods to control for confounding, selection bias, and other sources of bias in observational data as well as careful consideration to the vocabulary and differences in scientific jargon.

Second, we need to embrace and learn from heterogeneity rather than attempt to treat it as an unfortunate byproduct that we try to control for in our analyses. This will allow us to move from asking “What works?” to exploring “What works for whom under what conditions?” The variation both within and across health care systems provides a unique opportunity to learn which tools, practices, and organizational structures are most effective for achieving high quality.

Third, both CER and QIR need to tackle the challenge of spread and sustainability. CER that is never taken up into practice and QI findings that are not spread beyond their original setting both represent lost opportunities and wasted resources. As the field of QI reaches a tipping point, shifting the focus of both disciplines on how to spread knowledge generated in one setting to new settings and sustain it over time with high fidelity will be most beneficial.

Finally, CER and QIR need to pay continued attention to value in the broadest sense. The crisis in healthcare costs in the US requires that we examine which of our treatment alternatives and which of our quality improvement efforts represent the best value, considering an inclusive set of outcomes (including quality of life, patient satisfaction, and equity) and a comprehensive set of costs to patients and to society.

### Disclaimer

The opinions in this article represent those of the authors and not the official policy of the Department of Veterans Affairs.
